# Study on fracture development and progressive failure characteristics of downstream dam-type expansion tailings reservoir

**DOI:** 10.1038/s41598-022-25437-2

**Published:** 2022-12-01

**Authors:** Hongyue Zhang, Jiaxu Jin, Yihong Xu

**Affiliations:** 1 Liaoning Technology University, Fuxin, 123000 China; 2 Key Laboratory of Geotechnical and Underground Engineering of Ministry of Education, Tongji University, Shanghai, 200000 China; 3 Liaoning Key Laboratory of Mine Subsidence Disaster Prevention and Control, Fuxin, 123000 China; 4 Liaoning Provincial College of Communications, Shenyang, 11000 China; 5 Liaoning Bridge Safety Engineering Technology Innovation Center, Shenyang, 11000 China

**Keywords:** Civil engineering, Mineralogy

## Abstract

With the economic development and industrialization, the increasingly accumulated tailings ponds in China have become a great risk. Due to the difficulty of selecting proper site for a new reservoir in Yunnan, a sub-dam was built at the downstream original reservoir. This study explored the fracture development and progressive failure characteristics of the tailings reservoir area after capacity expansion based on a similarity experiment and the numerical simulation. The results showed that the primary cracks in the reservoir area were more than those at the top of the sub-dam. With the increase of the upper load, the primary cracks further developed and penetrated the whole sub-dam top, and the sub-cracks were then produced under the concentrate stress of the primary cracks. After the further development of the sub-cracks, the secondary cracks parallel to the primary cracks were formed on the outer slope of the sub-dam. The progressive failure of a tailings dam can be summarized as: the maximum shear stress was firstly generated at the toe of the slope or the top of the dam which then extended to the top of the sub-dam in the form of a curve and finally formed the failure surface by connecting with the primary fracture of the tensile plastic zone at the top of the dam. The study also found that in the process of tailings accumulation in the new reservoir area, tailings would form "back pressure slope protection" at the initial dam of the original reservoir, which not only effectively delayed the occurrence of shear failure, but also inhibited the generation and penetration of tensile plastic zone.

## Introduction

A tailings reservoir is composed of dams that intercept a valley mouth or enclose land, which is used to store tailings or other waste residues after metal or non-metal mines extraction. The tailings reservoir is an artificial debris flow hazard with high potential energy. The dam failure of tailings reservoir will cause irreparable catastrophic consequences^1–3^ and long-term environmental pollution^4–6^.

At present, many scholars have conducted comprehensive studies on the damage to tailings dams after earthquake and flood^7–11^, and have provided detailed theoretical references for the safe operation and failure warning mechanism of tailings dams under earthquake and flood conditions. There are also many studies on the failure mechanism and fracture development of the slope. For example, Xiaoyan et al.^12^ carried out similar experiments to study the progressive failure process of tailings slopes under concentrated water flow with different relative densities of tailings as variables. Tao et al.^13^ used temperature-sensitive material paraffin as the similar material to simulate the weak structural plane in the slope. The failure process of the slope was studied and divided into four stages: soil compaction stage, crack generation stage, crack propagation stage, and sliding surface penetration stage. Zheng et al.^14^ carried out an evaporation experiment of sedimentary tailings by using a constant temperature device, and analyzed the influence of initial concentration, particle size, exposed area, and sedimentary layer thickness on the evaporation process. Geng et al.^15^ studied the water content and the scale deformation characteristics of unsaturated tailings. The results showed that the microstructure deformation of unsaturated tailings can be divided into four stages: pore compression, elastic deformation, structural change, and compaction deepening. Lu^16–18^ analyzed the failure mechanism of a slope from a theoretical perspective and expounded on the progressive failure process of a slope in detail. In practical engineering, the failure modes of many slopes not only include shear failure, but also tensile-shear failure coexistence. Gao et al.^19^ established a linear yield criterion based on azimuth dispersion and considered the tensile failure criterion. The study showed that only considering shear failure may overestimate the slope stability. Wang et al.^20,21^ used the strain softening model to replace the ideal plastic model and proposed the strength reduction method and the slope vector sum analysis method considering tension-shear failure. The results showed that by considering the combined action of tension and shear force, the slope could obtain a more accurate safety factor, which was more in line with engineering practice. With the increase in tailings accumulation, the expansion of the original tailings has become an urgent problem to be solved. Fu-Sheng et al.^22^ calculated the infiltration line of a tailings dam slope, analyzed the dam body condition and stability safety factor after heightening and expansion, and proved that the elevation and expansion scheme of a tailings rockfill dam is safe and reliable. Wei et al.^23^ used the upper bend drainage system to analyze the successful cases of increasing and prolonging the service life of an existing tailings pond. Cao et al.^24^ carried out an experimental study on the effect of particle size distribution on the shear wave velocity (Vs) of unsaturated tailings. The results show that the soil water characteristic curve of unsaturated tailings sand is similar to that of other sands, and Vs is closely related to effective stress, particle size and void ratio.

After constructing sub-dam downstream the expanded tailings reservoir, a new reservoir will form downstream the original reservoir. With the accumulation of tailings in the new reservoir, the new and the original reservoir eventually merge. This expansion method is rarely adopted worldwide. Since the tailings capacity increases significantly after expansion, it poses great challenges to the safe operation of the tailings pond. Therefore, this paper used similar experiments and numerical analysis to analyze the fracture development and progressive failure process of the expansion tailings pond. The purpose of this study is to provide a reference for the operation and maintenance of the project, and to provide new methods and insights for similar projects to solve limitations of the new reservoir.

## Project profile

**Figure 1:**
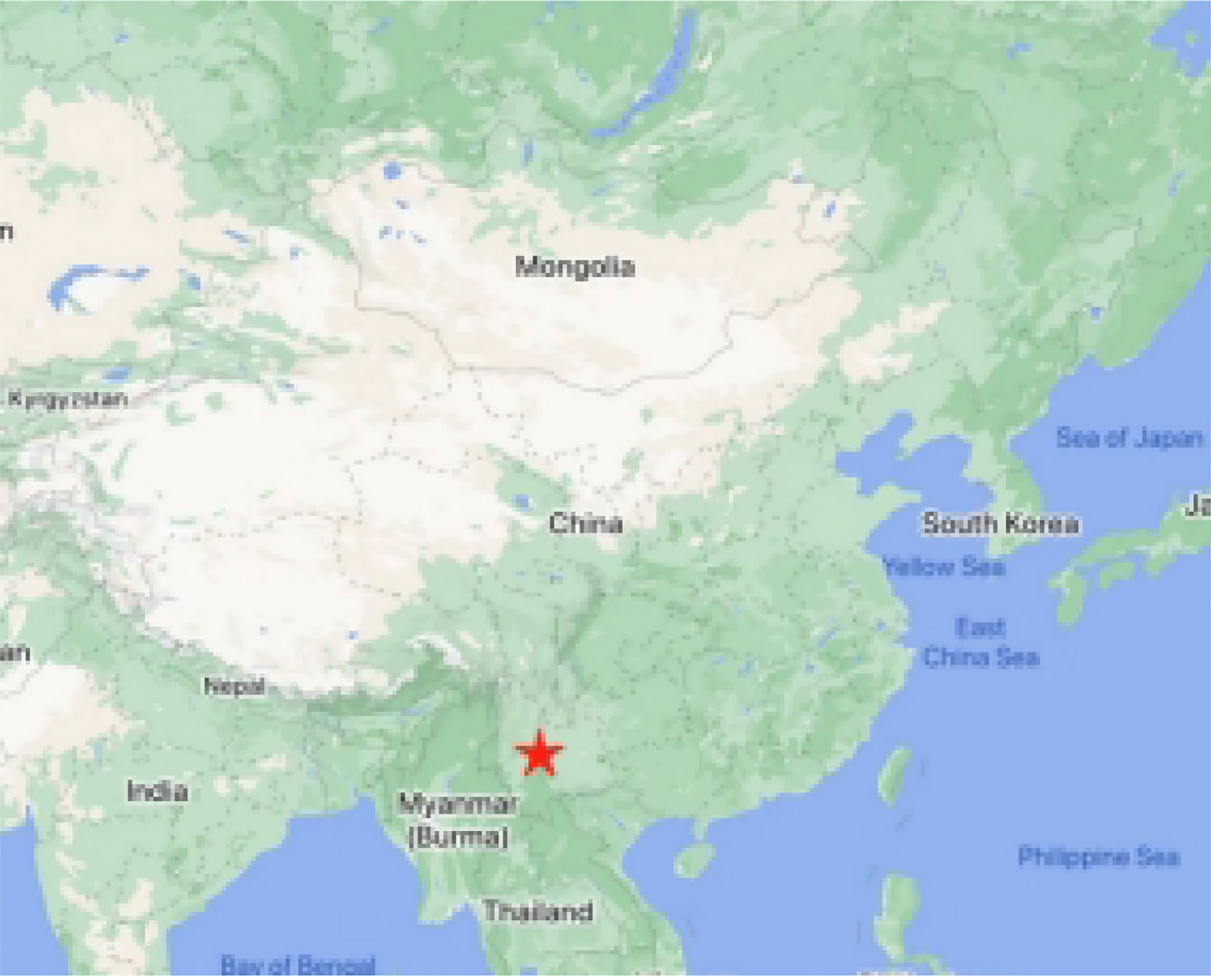
Geographical location of the tailings pond in Yunnan.

The working area is located on the western slope of the Ailao Mountains in Yuxi City, Yunnan Province, China. The geographical location is shown in Fig. 1. The initial dam of the expanded tailings pond is located at about 640.0 m downstream the original tailings dam site of Banmao Gully, which is at the same elevation of the design tailings pond. The proposed initial dam height is 64.0 m, the dam crest width is 5.0 m, the dam bottom width is 255.0 m, the dam crest length is 180.0 m, and the dam type is rockfill dam. The total storage capacity is 1757.3 million m^3^, and the effective storage capacity is 1521.9 million m^3^, which belongs to the second-class storage reservoir. The final accumulation elevation is 1260.0 m, the accumulation slope is 1:4.5, and its designed service life is 19.4 years.

1. The initial dam of the original tailings reservoir: a tailings reservoir was built in 2008 and went into service in June 2009. The initial dam is a rolling face rockfill dam, which is rolled by a mixture of weathered rock and soil in the tailing reservoir and block stone in the milling area. According to the measured data, the initial dam crest elevation is 1175.00 m, the dam bottom elevation is 1149 m, and the dam crest width is 3.00 m. A track is set up on the external dam slope, the elevation, the width, the upper slope ratio and the lower slope ratio of which is 1160.00 m, 1.50 m, 1:2.0 and 1:2.25, respectively, and the step width is 1.50 m; the slope-to-slope ratio of the internal dam is 1:1.75, and the elevation of the slope toe of the internal dam is 1152.05 m. The dam is in the southwest-northeast direction, and the crest length of the initial dam is 40.00 m. According to the field investigation, the initial dam operation so far shows that the dam is intact without leakage, cracking, or other deformation signs.
2. The original tailings dam: the dam is made from weathered soil on the bank slope, with the initial accumulation height of 26 m and the current total dam height is 99 m. The sub-dam is approximately parallel to the initial dam, with a length of 40–330 m, and the outer slope ratio of the sub-dam is 1:3.0. Drainage ditches have been built. The crest width of the sub-dam is 4.0 m, and the height of the sub-dam on the whole site is 4.0 m. The local area is compacted and most of them are loose. The silty clay is filled in the sub-dam, and there are long-term water level observation holes and saturation line observation holes on the sub-dam. According to the field investigation, there is no cracking deformation in the dam body.
3. The original tailings reservoir area: the dry beach length of the tailings reservoir is about 150–250 m, and the reservoir water depth is 0.2–1.5 m. Referring to the survey reports in 2012 and 2017, the water level of the reservoir area is mostly 12.8–17.5 m. The main deposits in the reservoir area are tailing silt and tailing silty clay.

## Testing models and methods

### Similarity conditions

To make the failure modeling test results credible and instructive, the geometric similarity, physical similarity, and motion similarity should meet the demands of the modeling test. The test material should also be selected according to its geometric similarity. However, due to the small size of the tailings sand material, it is difficult to find the material required to meet demands of the modeling test. In the similarity test, it is difficult to meet all the similarity criteria because the original project is a coupling of various complex fields. Therefore, when selecting the model sand, it is only necessary to meet the same objective law and the approximate similarity of the main physical properties, and it is not necessary to excessively criticize the strict similarity of a certain physical property^25,26^.

The similarity criteria of the modeling test are as follows:

1. Geometric similarity
  1 $$ \alpha_{L} = \frac{{L_{H} }}{{L_{M} }} = 1000 $$
2. Physical similarity
  2 $$ \alpha_{t} = \frac{{t_{H} }}{{t_{M} }} = \sqrt {\alpha_{L} } \approx 31.62 $$
3. Motion similarity
  3 $$ \alpha_{u} = \frac{{u_{H} }}{{u_{M} }} = \frac{{L_{H} /t_{H} }}{{L_{M} /t_{M} }} = \alpha_{L} \alpha_{t}^{ - 1} \approx 31.63 $$
  4 $$ \alpha_{a} = \frac{{a_{H} }}{{a_{M} }} = \frac{{u_{H} /t_{P} }}{{u_{M} /t_{M} }} = \alpha_{L} \alpha_{t}^{ - 2} \approx 1 $$
4. Gravity similarity
  5 $$ \alpha_{g} = \frac{{g_{H} }}{{g_{M} }} = 1 $$
  where H and M represent prototypes and models; *α*_*L*_ is length similarity ratio, *L*_*H*_ is prototype length, *L*_*M*_ is model length; *α*_*t*_ is the similarity time ratio between prototype and model, *t*_*H*_ is the prototype motion time, *t*_*M*_ is the model motion time; *α*_*u*_ and *α*_*a*_ are velocities and acceleration similarity ratio, *u*_*H*_ is prototype velocity, *u*_*M*_ is model velocity, *a*_*H*_ is prototype acceleration, *a*_*M*_ is model acceleration; αg is a gravity similarity ratio of 1.

### Experimental design

#### Loading method

In this experiment, the load of tailings pond was increased by gradually increasing the inclination angel of test box. In the modeling test, the roller was set below the test box, and the other end was connected with the uniform motor through a fine chain. During the test, the test box was lifted at a uniform speed, and the angle measuring device was placed on the side of the test box to measure the lifting angle. With the increase of inclination angle *Φ*, the internal shear stress of tailing sand in the test box gradually increased, and finally reached the failure state.

#### Test equipment and scheme

**Figure 2:**
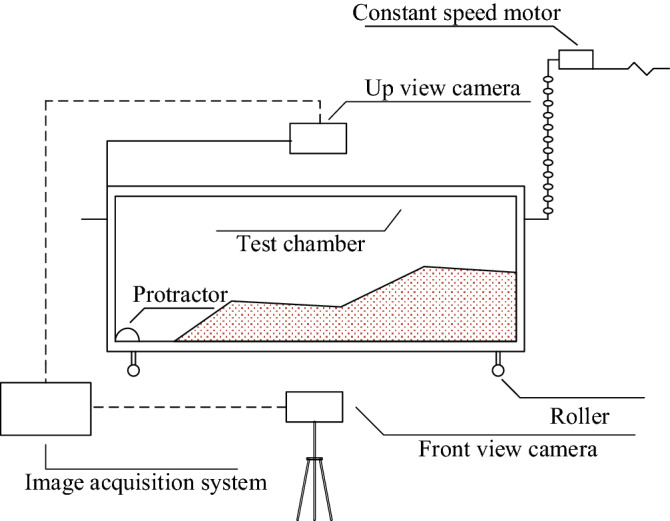
Test device diagram.

According to the test requirements, a test box with four sides of stainless-steel material and a front of tempered glass material was customized, and the size of the box was 80 cm × 35 cm × 20 cm. An industrial camera with a pixel count of 3 million was installed in the upper and front of the test box to record the experiment process. The real-time data was transmitted to the computer through the USB data connection line. The image acquisition resolution was 2048 × 1536, and the number of image acquisition frames was 1 FPS-15 FPS. The test device was shown in Fig. 2.

**Figure 3:**
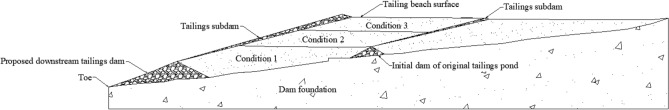
Test models of three working conditions.

In this experiment, three working conditions of a tailings reservoir in Yunnan after expansion were simulated: (1) the tailings beach surface of the new reservoir area reaches the top of the initial dam of the original reservoir pond (Condition 1), (2) the tailings beach surface of the new reservoir area rises to the midpoint of the sub-dam in the original reservoir area (Condition 2), and (3) the beach surface of the new reservoir area comes to the top of the sub-dam in the original reservoir area (Condition 3). After forming the tailings dam model, the dam body surface was compacted with a baffle. The schematic diagram of the working conditions is shown in Fig. 3. Under three working conditions, the tailings are accumulated layer by layer and each layer was compacted in order to simulate the actual working conditions. The load was increased by lifting the test device and for each time, the lifting angle was 1° and the static time was 3 min. The industrial camera recorded in real-time images during the lifting process. The raw tailings were used as the test material, with a dry density of 1.92 g/cm^3^ and a moisture content of 12.3%. The particle composition is shown in Table 1.

**Table 1:** Composition of tailings particles.

Content ratio	Particle composition/mm							
	> 2	2–1	1–0.5	0.5–0.25	0.25–0.075	0.075–0.045	0.045–0.005	< 0.005
Retention sieve quality/%	2.14	1.24	9.55	28.56	40.57	6.67	4.02	7.25
Cumulative content/%	100	97.86	96.62	87.07	58.51	17.94	11.27	7.25

### Quantitative analysis of fractures

**Figure 4:**
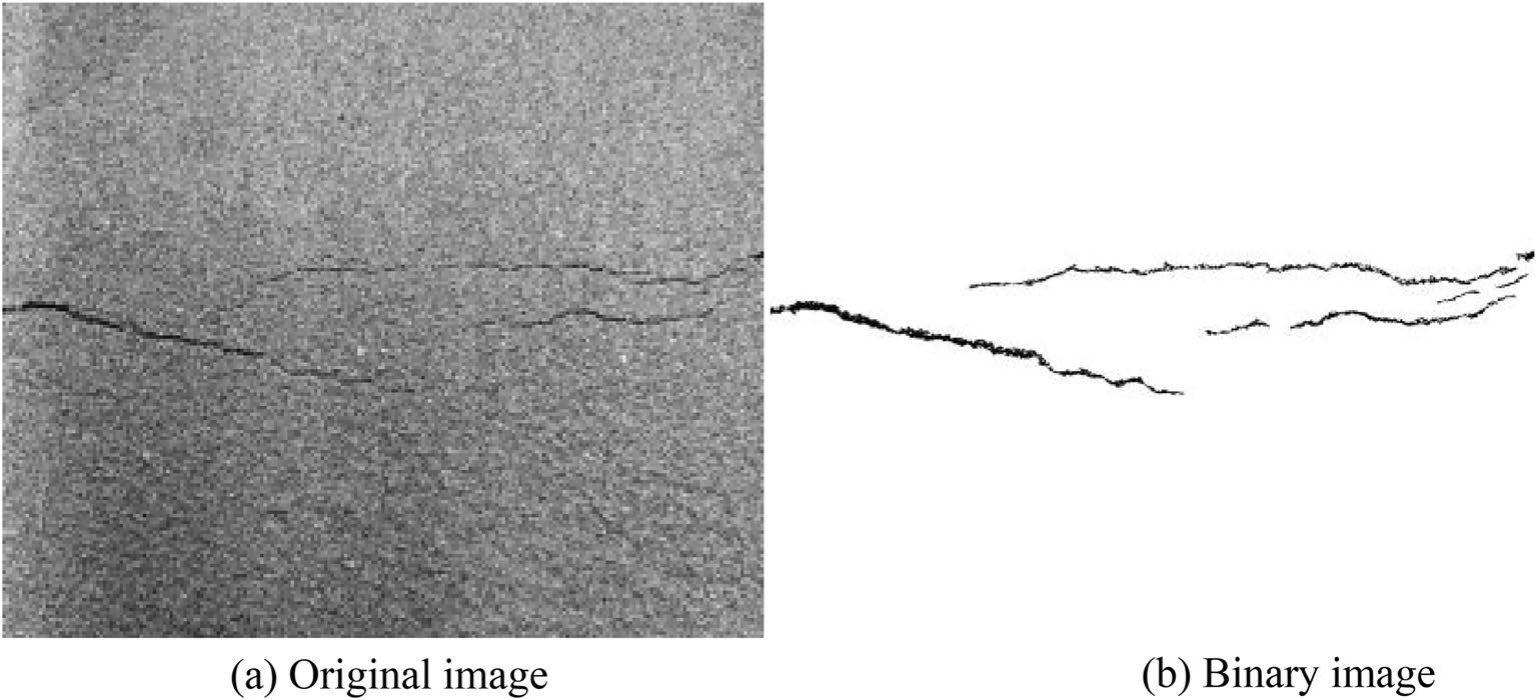
Binarization of tailing sand fracture image.

The obtained images were grayed and threshold values were segmented by MATLAB software, as shown in Fig. 4. The fracture network can be identified and the geometric characteristics of the fracture can be calculated by the "regionprop" function of MATLAB software. In this experiment, the maximum long axis length and the maximum short axis length of the tailings fracture (the maximum long axis and the short axis length of the ellipse with the same standard second-order central distance in the region) were calculated. Since MATLAB software calculated the geometric characteristics of cracks in the image based on pixels, Photoshop software was used to process the image into a binary image with the same resolution of 220 dpi × 220 dpi before the calculation, so the width of a pixel was 0.11 mm.

The boundary of the test box can hinder or even change the development of cracks to a certain extent. When the tailing in the middle part of the box had a downward trend, the tailing at the wall of the device was still in a static state, resulting in the concentration of shear stress at the tip of the crack, under which condition the crack changed from the tensile stress to the combined action of tensile stress and shear stress. The direction of crack growth and development were also changed. Therefore, in this paper, the image boundary part was cut off during the image processing, and only the middle part of the image was studied.

## Test results and analysis

### Fracture development law in the reservoir area

**Figure 5:**
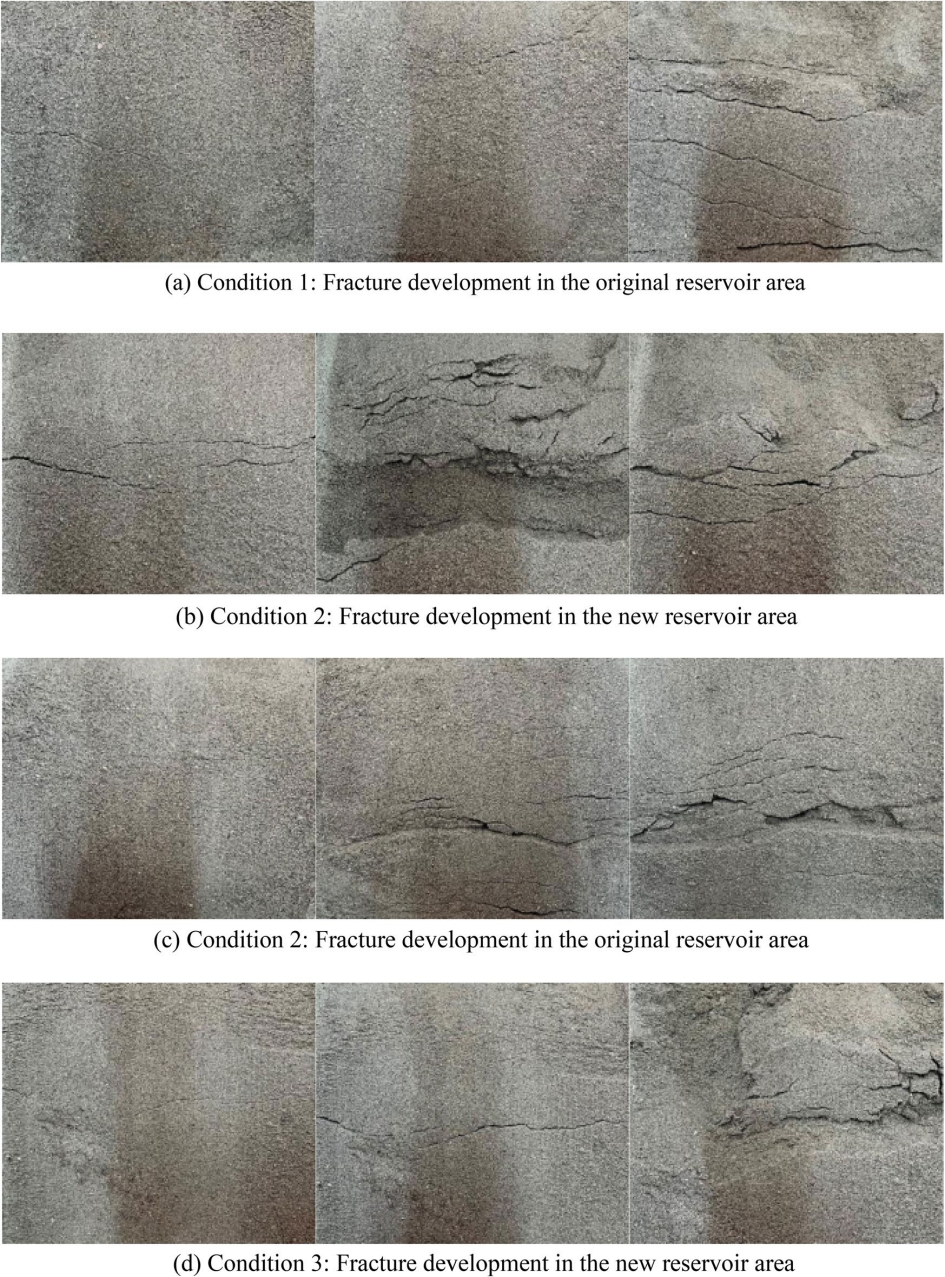
Fracture development map under three working conditions.

Figure 5 (although the binary image can clearly reflect the development characteristics of fractures, it will cover the differential settlement in the new and original reservoir areas, so the binary image is not used) represents fracture development images under three working conditions. In condition 1, the new reservoir area was covered by sliding tailings when the original reservoir dam was destroyed, and the fracture development of the new reservoir area were not observed. In condition 3, the new and original tailings became a whole, so there was no fracture development in the original reservoir area.

According to the fracture development diagrams of three working conditions, with the increase of the inclination angle of the device, the gravity component of the tailings at the top of the dam increased gradually along the slope direction, and the primary cracks began to appear in the reservoir area. The length, width, and the number of primary cracks were not the same under different working conditions. Under Condition 1, the primary cracks in the original reservoir area initiated at the top of the dam. The cracks grew from the outer boundary to the reservoir area but the further development of cracks was not obvious. With the increase of load, the primary cracks further developed and penetrated the top of the sub-dam. At the same time, the sub-cracks were derived from the stress concentration of the primary cracks, and the sub-cracks grew vertically from the primary cracks to the outer slope of the sub-dam. After the further development of the sub-cracks, the secondary cracks were formed on the outer slope of the sub-dam, which were approximately parallel to the primary cracks. With the emergence of secondary cracks, a small range of landslides occurred on the outer slope of the sub-dam. As the inclination angle continued to increase, collapse failure took place at the top of the sub-dam where the primary fractures were initiated. In the Condition 1, the fracture development and the failure form of the original reservoir area are 'concave' while in the Condition 2, it shows the opposite 'convex' shape. The author believes that the tailings level in the new reservoir area is low, which cannot provide an effective support for the original reservoir area. With the generation of primary and secondary cracks on the surface of the dam body, the retrogressive failure begins to appear. The failure surface is distributed in a 'concave' shape. The collapsed sliding body extends to the reservoir area in a step shape, which eventually leads to the overall failure of the reservoir area. With the increase of the accumulation of tailings in the new reservoir area, an effective pressure slope is formed at the original reservoir area, and the failure form of the reservoir area is transformed into push failure, so the fracture development form and the failure surface are 'convex'.

Under the Condition 2, the new reservoir area generated cracks before the original reservoir area, and the location and development direction of primary cracks were consistent with those of the original reservoir area under the Condition 1. But differently, the primary cracks in the new reservoir area under this condition were distributed in parallel with multiple branches and almost connected through the whole dam crest. With the increase of the overlying load, the secondary cracks were distributed in a multi-level parallel manner on the slope outside the dam. The generation of primary cracks, sub-cracks, and secondary cracks in the original reservoir in the Condition 2 was roughly the same as that in the original reservoir under the Condition 1. The difference was that the width of primary cracks continued to increase with the increase of load, and the tailings at the edge of cracks deteriorated and collapsed, but the overall sub-dam remained stable. Under the Condition 3, due to the increase in tailings accumulation height, the local collapse failure began to occur after the initiation of primary cracks. With the increase of the inclination angle of the experimental device, the load on the top of the dam increased, and the tailings dam showed a rapid landslide.

### Fractal characteristics of tailings reservoir fractures

The fractal theory is a good tool for describing irregularity and self-similarity. In recent years, it has been widely used in soil research. The results showed that soil has good self-similarity^27–31^. Therefore, the fractal theory was used to describe the fracture characteristics of the tailings pond.

The research object of this paper is fractures in the reservoir area, and the fractal theory was used to quantitatively study the fractures in the reservoir area, while the box-counting dimension method^32^ was used to calculate the fractal dimension of the fractured image in the reservoir area with MATLAB software. Let *F* be an arbitrary nonempty bounded subset of *R*^*n*^, *N*_*δ*_(*F*) be the minimum set with a maximum diameter of *δ* and a covering of *F*, then the lower and upper box dimensions of *F* can be defined as:

6 $$ \underline{\dim }_{B} F = \mathop {\underline{\lim } }\limits_{\delta \to 0} \frac{{\log N_{\delta } (F)}}{ - \log \delta } $$

7 $$ \overline{\dim }_{B} F = \mathop {\overline{\lim } }\limits_{\delta \to 0} \frac{{\log N_{\delta } (F)}}{ - \log \delta } $$

If these two values are equal, they are called box-counting dimensions of *F*, denoted as:

8 $$ \dim_{B} F = \mathop {\lim }\limits_{\delta \to 0} \frac{{\log N_{\delta } (F)}}{ - \log \delta } $$

**Figure 6:**
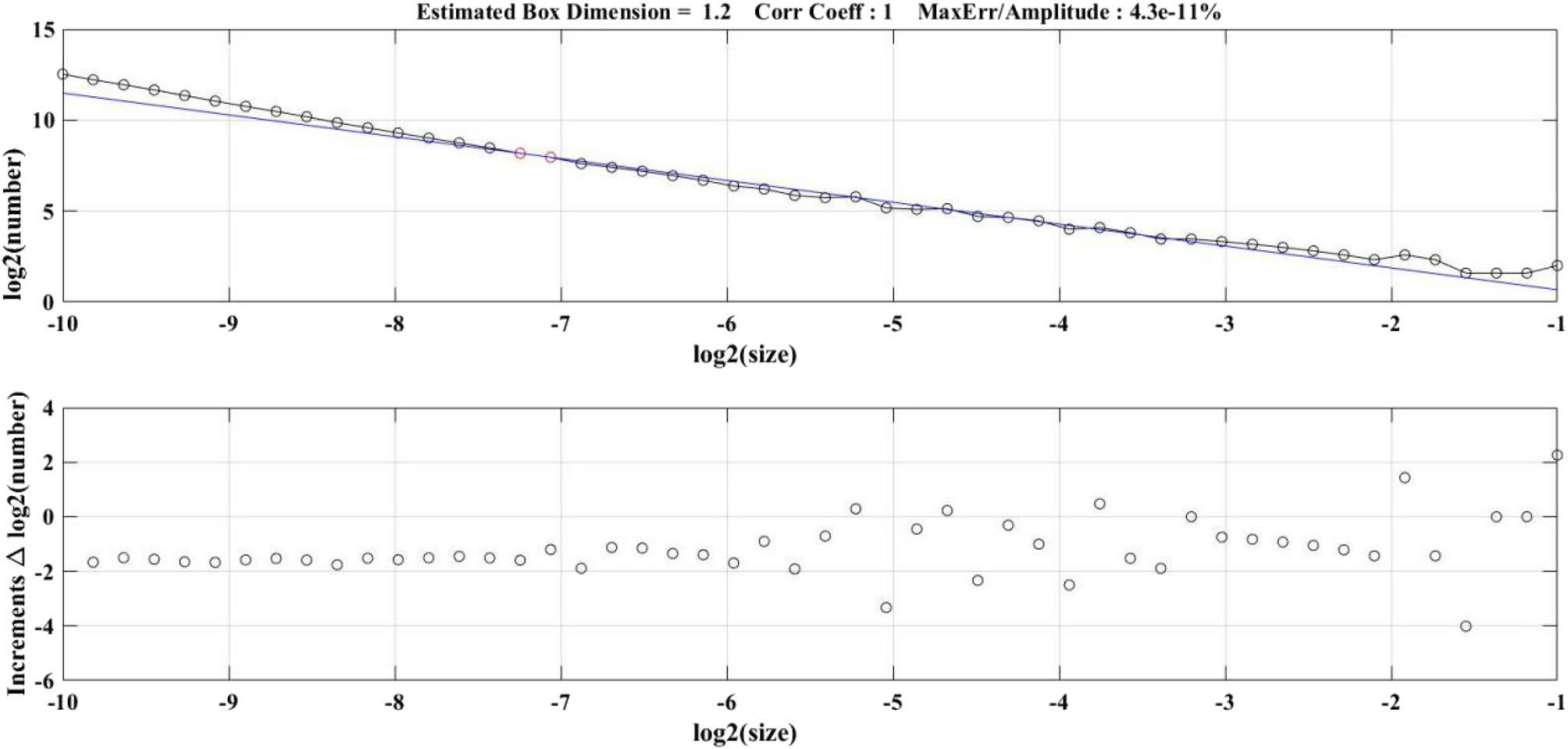
Condition 1: Log*N*_*δ*_(*F*) and log*δ* logarithm images of primary fractures in the original reservoir area.

**Figure 7:**
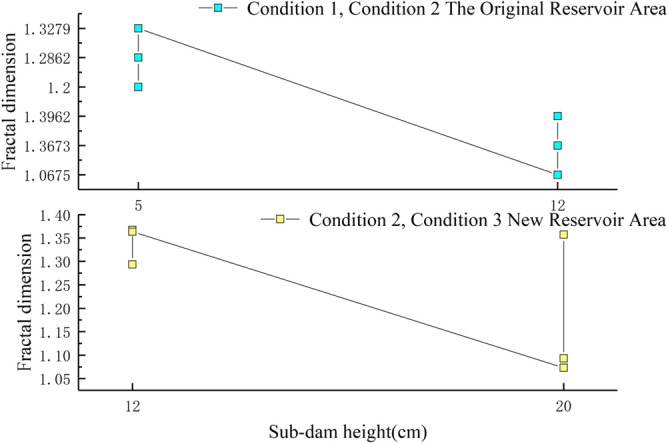
Fractal dimension of new and original reservoir fractures under three working conditions.

Figure 6 shows log*N*_*δ*_(*F*) and log*δ* logarithmic images of primary fractures in the original reservoir area, and the least square method was used to fit them. The fractal dimension of fractures in the original reservoir area was 1.2, the correlation coefficient *R*^2^ was close to 1, and the maximum error was 4.3e−11%, which showed that the fractures in the original reservoir area have obvious statistical self-similarity. Figure 7 shows the variation of fracture fractal dimension with the increase of dam height in the new and original reservoir areas under three conditions (the original reservoir dam height is from 5 to 12 cm and the new reservoir dam height is from 12 to 20 cm). When the primary fractures appeared in the new and original reservoir areas under three conditions, the fracture fractal dimension was close to 1, indicating that the shape of primary fractures was similar to a straight line, and with the further development of fractures, the sub-fractures and secondary fractures led to an increase of fractal dimension. Under the Condition 2, the fracture development of the original reservoir area was later than that of the Condition 1, and the fracture development was more regular. This paper analyzed the back pressure slope formed at the original reservoir area with the gradual accumulation of tailings in the new reservoir area, which enhanced the stability of the original reservoir area.

### The influence of accumulation height on the geometric properties of primary fractures in a new reservoir area

**Figure 8:**
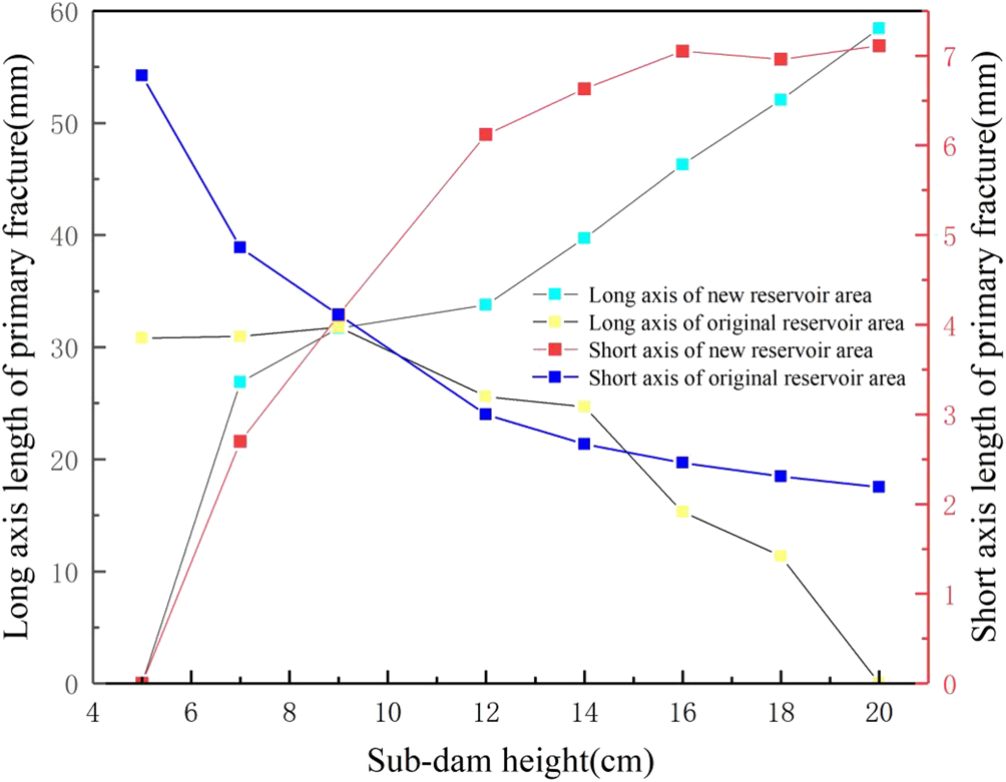
Accumulation height of the new reservoir area and geometric characteristics of primary fractures in the new and original reservoir areas.

To explore the relative relationship between the geometric characteristics of primary fractures in the new and original reservoir areas and the increase of tailings accumulation height in the new reservoir area, five groups of tailings accumulation heights in different new reservoir areas were added under three typical working conditions, and each group was tested three times. The average length of primary fractures and short axis length calculated by MATLAB software were taken. The stacking height of the new reservoir area and the geometric characteristics of the primary fractures in the new and original reservoir areas are shown in Fig. 8. With the increase of the accumulation height of the new reservoir area, the length of the long and short axes of the primary fractures in the new reservoir area showed an overall increasing trend, and the increase rate of the short axis was significantly higher than that of the long axis. The increase rate of the long axis was 117.4%, and that of the short axis was 163.3%, indicating that with the accumulation of tailings, the length and width of the primary fractures in the new reservoir area significantly increased, and the increase rate of the width was higher. The long and short axes of primary fractures in the original reservoir area showed a decreasing trend. The long axis decreased from 30.81 to 11.37 mm, with a decrease of 63.1%. The short axis decreased from 6.78 to 2.19, with a decrease of 67.7%. There was little difference in the decrease rate. The decreasing trend of the long and short axes of the primary cracks in the original reservoir area can be verified with the "back pressure slope protection" below. It is precisely the increased height of the accumulated tailings in the new reservoir area that inhibits the initiation and development of the cracks in the original reservoir area. However, corresponding reinforcement measures were not taken in the new reservoir area. With the accumulation of tailings, the trend of fracture development is more intense. Therefore, it is suggested that the tailings reservoir with downstream dam expansion should be used to reinforce the dam body in the new reservoir area, which can effectively improve the stability of the tailings reservoir.

## Progressive failure characteristics of the tailings pond

In recent years, many scholars have found that there are theoretical defects in the design of modern slope engineering practice. Specifically, the slope instability is a composite failure process rather than the shear single factor represented by traditional classical geotechnical mechanics. In practical engineering, slope instability is the result of combined action of tension and shear force. Therefore, when analyzing the stability of the slope, if only the shear failure is considered, the calculated safety factor will be overestimated to a large extent, and thus the stability of the slope may be wrongly evaluated^33–35^.

In this study, the FLAC3D software was used to simulate the three working conditions of the expansion tailings pond, and the diagram of the working condition is shown in Fig. 3. The three principal stresses of the composite shear-tension failure criterion in FLAC3D meet:

9 $$ \sigma_{1} \le \sigma_{2} \le \sigma_{3} $$

The shear failure criterion is defined as:

10 $$ \left. \begin{aligned} & f_{s} = \sigma_{1} - \sigma_{3} N_{\varphi } + 2c\sqrt {N_{\varphi } } \hfill \\ & N_{\varphi } = \frac{1 + \sin \varphi }{{1 - \sin \varphi }} \hfill \\ \end{aligned} \right\} $$

The tension failure criterion is defined as:

11 $$ \left. \begin{aligned} & f_{t} = \sigma_{3} - \sigma_{t} \hfill \\ & \sigma_{t} = \min (\sigma_{0} ,\frac{c}{\tan \varphi }) \hfill \\ \end{aligned} \right\} $$

where *φ* is internal friction angle, °; *c* is cohesion, kPa; *σ*_*t*_ is tensile strength, kPa; *σ*_*0*_ is the initial tensile strength of soil, kPa.

The strength parameters of each partition in the numerical model are listed in Table 2. In the calculation process, the Mohr–Coulomb model considering tension-shear is used for the tailing sand, and the ideal elastic model is employed for the tailing dam and dam foundation.

**Table 2:** Physical parameters of partition materials.

Material name	E/MPa	μ	Volumetric weight/(kN m^−3^)	Friction angle/(°)	Cohesion/kPa	Dilatancy angle/(°)	Tensile strength/kPa
Tailings	24	0.4	17.9	17.2	10.6	0	50

Material name	Volumetric weight/(kN m^−3^)	K/GPa	G/GPa				
Tailings dam	22.0	20.3	4				
Dam foundation	27.1	26.8	7				

### Tensile failure characteristics

**Figure 9:**
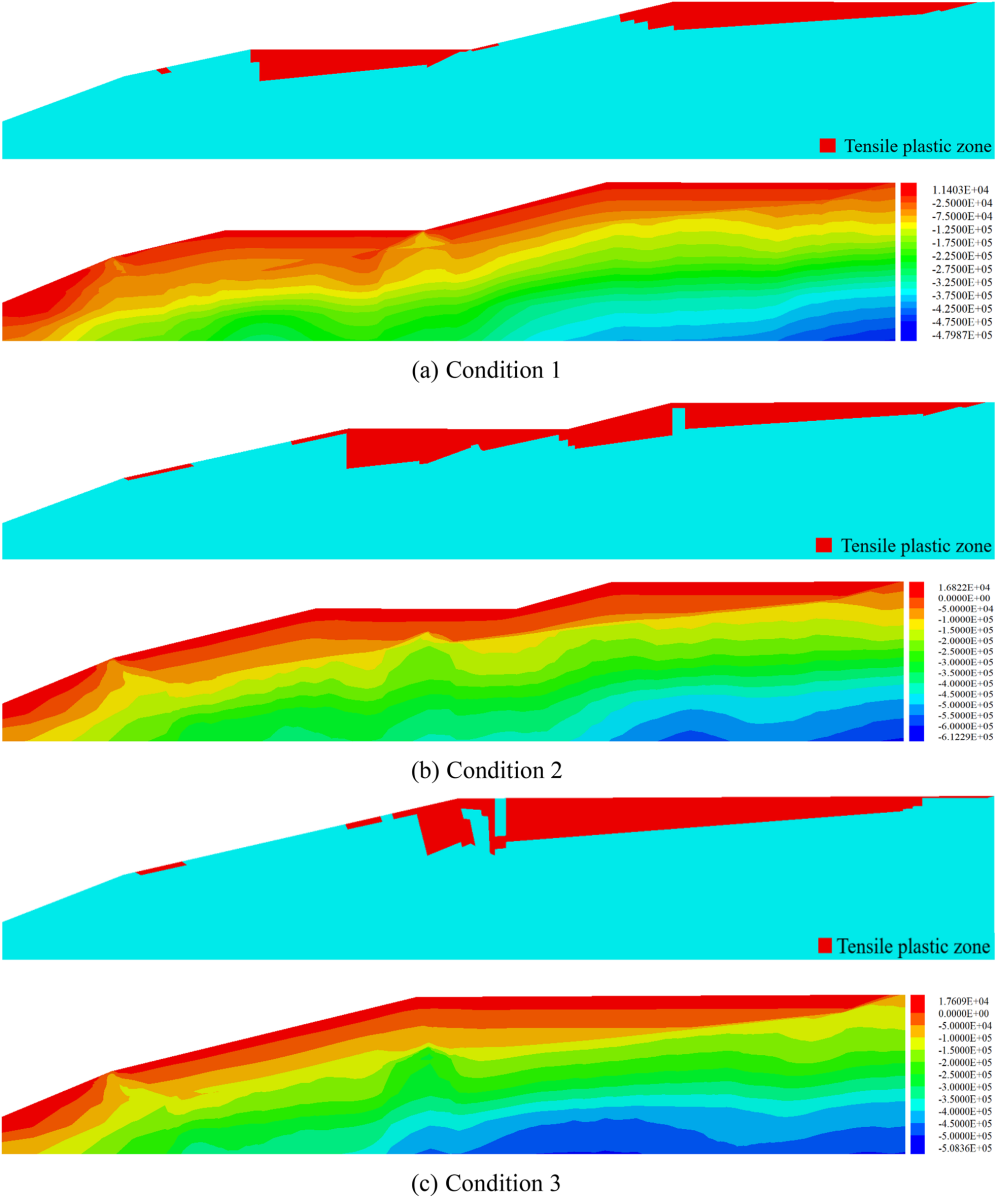
Distribution of tension plastic zones and the minimum principal stress in new and original reservoir areas.

The upper side of Fig. 9 is the distribution map of tensile plastic zone in the new and original reservoir areas, and the right side is the distribution map of minimum principal stress. It can be seen from the diagram that at the initial stage of the expansion of the tailings pond, a large area of red tensile plastic zone appeared in the new and original reservoir areas. The plastic zone was concentrated at the top of the sub-dam and extended to the reservoir area. The distribution of the minimum principal stress showed that there was a tensile stress concentration zone on the surface and shallow layer of the reservoir area, which led to the formation of a plastic zone. The plastic zone near the sub-dam was deep, so reservoir instability occurred mostly at the top of the sub-dam. With the accumulation of tailings in the new reservoir, the initial dam and its overlying tailings in the original reservoir area can prevent the connection between the dam slope in the original reservoir area and the tensile plastic zone in the reservoir area to a certain extent. In other words, it prevented the formation of the tensile plastic zone at the top of the sub-dam. The "back pressure slope protection" can not only effectively delay the occurrence of shear failure but also inhibit the generation and connection of a tensile plastic zone. With the accumulation of tailings in the new reservoir area, the tensile stress on the surface and shallow layer of the reservoir area gradually increased, and the plastic zone gradually deepened. Therefore, with the accumulation of tailings, the surface of the reservoir area was more likely to produce primary cracks. When the tailing sand in the new reservoir area accumulated to the top of the dam in the original reservoir area, i.e., in the three working conditions, the connected region of the inverse plastic zone appeared. The analysis in this paper showed that when the tailing sand accumulated to a certain height, the original initial dam in the lower part of the sand body produced a "wedge" stress on the tailing sand, resulting in the concentration of tensile stress in the upper region, while the upper and lower slopes of the dam body played a supporting role in the tailing sand and reduced its tensile stress level. Therefore, when the tailing sand accumulated at the top of the dam in the original reservoir area, more attention should be paid to the observation of the tensile stress concentration area at the top of the dam to prevent the occurrence of primary cracks caused by tensile stress and to the further impact on the stability of the reservoir area.

### Tension-shear failure

**Figure 10:**
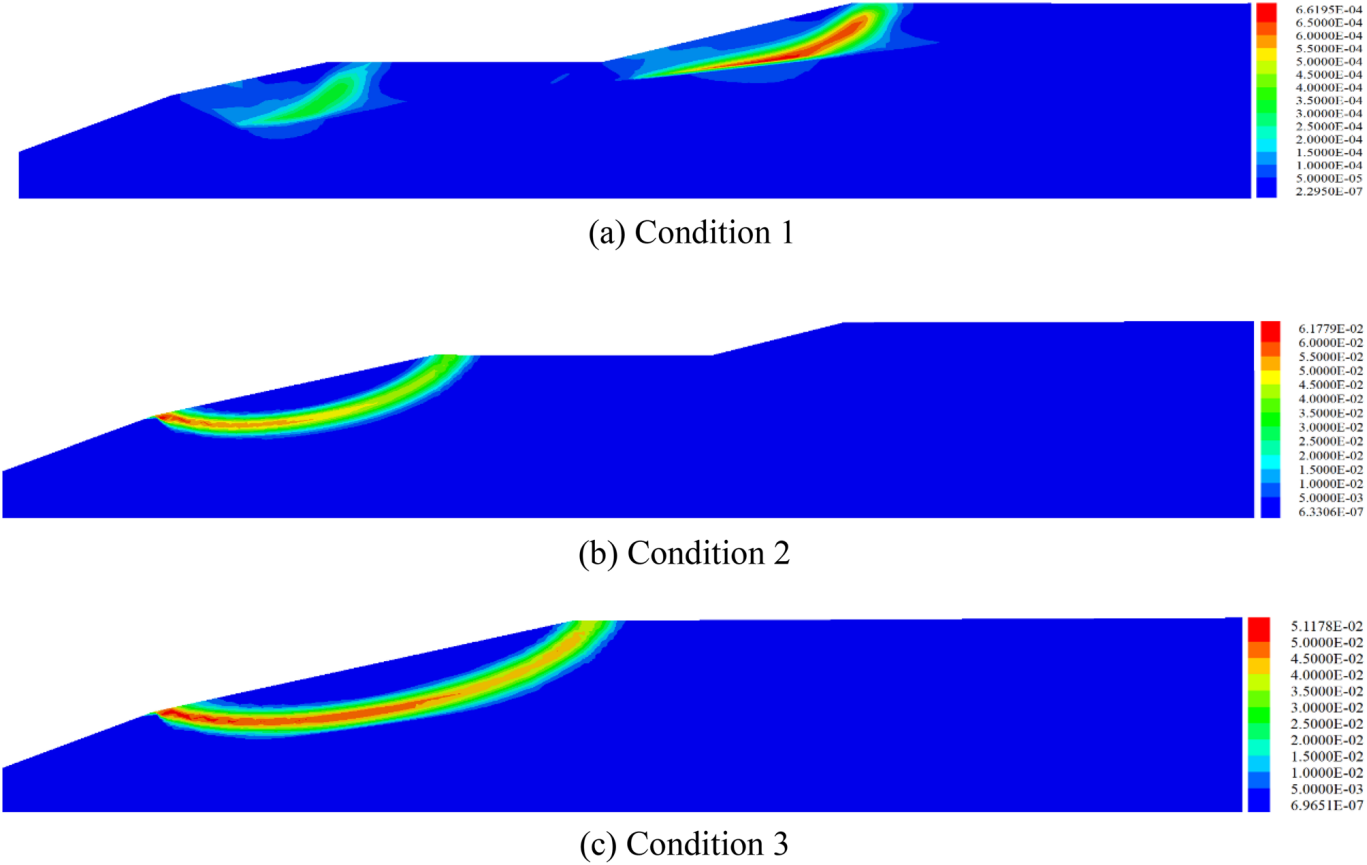
Distribution of the maximum shear stress in the reservoir area.

Combined with the distribution characteristics of maximum shear stress and tensile plastic zone in reservoir area in Fig. 10, the progressive failure of the tailing dam can be characterized as: (1) the maximum shear stress is generated at the slope toe or the initial dam crest, (2) and then extends to the crest of the sub-dam in the form of a curve, and (3) finally forms the failure surface by connecting with the primary fracture in the tensile plastic zone at the crest. From the maximum shear stress distribution, it can also be seen that under Condition 1, the instability failure of the reservoir area is the result of combined action of shear and tension failure. With the accumulation of tailings in the new reservoir area, the shear failure of the reservoir area will gradually become the dominant type. This is because, in the early stage of tailings discharge, the height of the reservoir area is low, the slope toe is small, and the anti-sliding force of the tailings in the reservoir is much larger than the sliding force. Therefore, the shear effect is weak, and the tensile strength of the tailings become low, so it is prone to produce a tensile plastic zone, which leads to the generation of primary cracks. With the gradual increase in the height of the tailings, the sliding force of the tailings in the reservoir will increase sharply, and the **"**reverse pressure slope protection" and "wedge stress" will be generated. The tensile stress will be weakened and the shear effect be more prominent.

## Discussion

In this paper, the fracture development in the reservoir area of the new tailings dam were studied by the similar test and numerical simulation method. The results showed that the failure in the reservoir area resulted from the combined action of tension and shear force. The typical failure process is summarized as: (1) the tensile plastic zone firstly appeared at the top of the new and original reservoir area; (2) the primary cracks were induced and initiated; (3) the cracks were further developed; (4) the sub cracks and the secondary cracks generated and the small landslide occurred; (5) the uneven settlement took place at the sub dam crest; and (6) the reservoir area lost stability eventually. Specifically, with the increase of the dip angle of the reservoir area, the tensile plastic zone appeared at the top of the sub dam in the new and original reservoir areas, which led to the significant reduction in the tensile strength of the soil. With the further expansion of the plastic zone, the primary cracks were initiated at the top of the dam. When the dip angle was enlarged, the primary cracks were further developed and run through the top of the dam. The sub-cracks were consequently induced under the concentrated stress of the primary cracks. The sub-cracks grew perpendicularly to the primary cracks and extended to the outer slope of the sub-dam. Afterwards, secondary fissures appeared, and local landslides took place on the outer slope of the sub-dam. The further increase in the dip angle caused the uneven settlement and collapse at the top of the dam where the primary cracks were initiated. As the damage became more severe, the reservoir area lost stability and landslide occurred as a result. The accumulated tailings sand in the new reservoir area served as a protective slope at the initial dam of the original reservoir area, providing a back pressure that inhibits the development of cracks in the original reservoir area and effectively improved the stability of the original reservoir. In such situation, the failure mode of the original reservoir area changed from retrogressive failure to failure caused by thrust load. When the height of accumulated tailings sand elevated to the midpoint of the sub dam in the original reservoir area, the plastic zone gradually deepened due to the increase of tensile stress on the surface and shallow layer of the reservoir area. Consequently, primary cracks were induced on the surface of the reservoir area. With the further accumulation of the tailings sand in the new reservoir area, the new and original reservoir areas became a whole, and the sliding force of the tailings in the reservoir area increased greatly. The generation of reverse pressure slope protection and wedge stress alleviated the tensile failure but enhanced the shear failure. This study can provide a basis for the operation and maintenance of the project, and provide a new solution for the expansion of similar projects limited by the location of the new reservoir area.

Nevertheless, several limitations of this study should be noted. In the similar test, the gravity component of the sand body along the slope direction was increased by increasing the lifting angle of the test box. The fracture development and failure process in the reservoir area were emphasized, but the relationship between the lifting angle and the fracture development and instability failure in the reservoir area were not considered. Besides, the size of the test box was relatively small, and thus the fracture development was studied in a relatively small zone. This may fail to represent the real failure process comprehensively. During the test, only the cracks on the surface were described, and the longitudinal development of the cracks was not explained and analyzed. The longitudinal development of the cracks also has a significant impact on the failure mode of the tailings reservoir. The fractal dimension of fractures at specific stages under different working conditions was calculated according to the obtained images, but the dynamic fractal dimension characteristics in fracture development were not obtained.

## Conclusions

In this paper, the fracture development and progressive failure characteristics of the downstream dam expansion tailings reservoir are studied by similarity experiments and numerical simulation. The results are as follows:

1. Under three typical conditions, the primary cracks in the new and original reservoir areas were greater than those at the top of the sub-dam. With the increase of the load, the primary cracks further developed and penetrated the top of the sub-dam. At the same time, the sub-cracks were derived from the stress concentration of the primary cracks. After the further development of the sub-cracks, the secondary cracks near the primary cracks were formed on the outer slope of the sub-dam.
2. The geometric characteristics of primary fractures in the reservoir area were linear. With the generation of sub-fractures and secondary fractures, the fractal dimension of fractures in the reservoir area increased, and the fractures developed from approximately linear development to dendritic development.
3. During the early stages of the expanded tailings reservoir's failure, a large area of tensile plastic zone appeared in the new and original reservoir areas, and the plastic zone was concentrated at the top of the dam and extended to the reservoir area; the emergence of "back pressure slope protection" can not only effectively delay the occurrence of shear failure but also inhibit the generation and penetration of tensile plastic zone.
4. When the tailing sand accumulated to a certain height, the original primary dam in the lower part of the sand body produced "wedge" stress on the tailing sand, resulting in the concentration of tensile stress in the upper region, while the upper and lower slopes of the dam body played a supporting role in the tailing sand and reduced its tensile stress level. Therefore, when the tailing reservoir adopted this expansion method, more attention should be paid to the tensile stress concentration area at the dam crest of the dam to prevent the occurrence of primary cracks caused by tensile stress and the impact on the stability of the reservoir area.
5. The progressive failure of a tailings dam can be characterized as: (1) the maximum shear stress is generated at the slope toe or the initial dam crest, (2) then extends to the crest of the sub-dam in the form of a curve, and (3) finally forms the failure surface by connecting with the primary fracture in the tensile plastic zone at the dam crest. Under the condition 1 (when the tailings beach surface of the new reservoir area rises to the top of the initial dam of the original reservoir area), the instability failure of the reservoir area is subjected to the combined action of shear-tension failure, and with the accumulation of tailings in the new reservoir area, the shear failure gradually becomes the dominant failure type.

## Data Availability

All data supporting the findings in this study are available from the corresponding author upon reasonable request.
